# A Stress-Neuroendocrine-Myeloid Inflammation Axis Is Associated with the Progression of Ménière's Disease

**DOI:** 10.1016/j.bbih.2026.101193

**Published:** 2026-02-06

**Authors:** Xiaofei Li, Na Zhang, Yongdong Song, Tongtong Zhang, Yafeng Lyu, Huirong Jian, Yawei Li, Jing Wang, Wenjuan Li, Yinghui Hu, Zhaomin Fan, Na Li, Daogong Zhang, Haibo Wang

**Affiliations:** a Department of Otolaryngology-Head and Neck Surgery, Shandong Provincial ENT Hospital, Shandong University, Jinan, Shandong, China; b Shandong Provincial Vertigo & Dizziness Medical Center, Jinan, Shandong, China; c Shandong Medical Health Key Laboratory of Vertigo & Vestibular Medicine, Jinan, Shandong, China; d Shandong Institute of Otorhinolaryngology, Jinan, Shandong, China

**Keywords:** Ménière's disease, Myeloid cells, β-adrenergic, Inflammation, Stress

## Abstract

**Background:**

Ménière disease (MD) is a chronic inner ear disorder of unknown etiology. Although an immune-inflammatory link is suspected, the upstream triggers and cellular mechanisms connecting psychosocial stress to inner ear pathology remain poorly defined. This study aimed to investigate the role of stress-related, myeloid cell-derived inflammation in the progression of MD.

**Methods:**

This multi-cohort study involved 384 MD patients (62.5% female); and 138 healthy controls (HCs) (46.4% female). Perceived stress was evaluated in 110 MD patients and 65 HCs. Transcriptional profiles were characterized using RNA sequencing on peripheral whole blood (4 MD, 6 HC) and on sorted CD11b + myeloid cells versus CD11b-non-myeloid cells (8 MD, 6 HC). In a larger cross-sectional cohort (239 MD patients, 35 HCs), the association between serum inflammatory cytokines and audio-vestibular function was examined, with a subset (n = 42) followed up. Finally, in vitro experiments explored the regulatory effects of catecholamines and glucocorticoids on myeloid cells isolated from 23 MD patients and 26 HCs.

**Results:**

MD patients reported significantly higher levels of perceived stress compared to controls. RNA sequencing of peripheral blood revealed a distinct pro-inflammatory transcriptional signature in MD patients. Further analysis identified CD11b + myeloid cells as the primary source of this inflammation, showing significant upregulation of pro-inflammatory genes. Clinically, elevated serum levels of the myeloid-derived cytokine G-CSF were associated with poorer baseline audio-vestibular function, and a follow-up increase in G-CSF levels correlated with functional deterioration. Mechanistically, MD patients exhibited elevated plasma norepinephrine and increased β1-adrenergic receptor mRNA in myeloid cells. In vitro, blockade of the β-adrenergic-cAMP pathway attenuated pro-inflammatory cytokine production in these cells. Although systemic cortisol levels remained unchanged, the glucocorticoid receptor sensitivity of myeloid cells in MD patients was altered.

**Conclusion:**

Our study elucidates a potential pathophysiological axis in MD, where perceived psychosocial stress is linked to sympathetic nervous system overactivity. This, in turn, primes circulating myeloid cells for a pro-inflammatory response via the β-adrenergic pathway. The resultant systemic inflammation is closely associated with the severity and progression of audio-vestibular dysfunction. These findings suggest that targeting the stress-neuroendocrine-immune interface may offer novel therapeutic strategies for MD.

## 1 Introduction

Ménière's disease (MD) is a chronic inner ear disorder of unknown etiology, characterized by episodic vertigo, fluctuating sensorineural hearing loss, tinnitus, and aural fullness (Lopez-et al., 2015). Its prevalence ranges from 17 to 513 per 100,000 individuals (Nakashima et al., 2016). While endolymphatic hydrops is a common pathological hallmark, the precise mechanisms driving this chronic condition remain elusive.

Dysregulation of the immune system has long been implicated in the pathogenesis of MD. This is supported by the phenotypic overlap between MD and autoimmune or allergic conditions, and the therapeutic efficacy of corticosteroids in managing vertigo and preserving hearing in MD patients (Derebery and Berliner, 2010; Gazquez et al., 2011; Webster et al., 2023). Several studies have reported elevated levels of inflammatory markers and heightened inflammatory responses in individuals with MD (Flook et al., 2023; Frejo et al., 2022; Zhang et al., 2023). However, a comprehensive understanding of the molecular underpinnings of systemic immune alterations in MD, particularly the upstream regulatory factors, the key cellular players, and their link to disease progression, is still lacking.

Clinical observations and epidemiological data suggest a strong association between psycho-behavioral stressors—such as sleep deprivation, depression, fatigue, anxiety, and trauma—and the onset or exacerbation of MD symptoms (Takahashi et al., 2005; Tyrrell et al., 2015; Yeo et al., 2018). This raises a critical question: how do these psychosocial factors potentially impact inner ear function via the immune-inflammatory pathway? Chronic psychosocial stress is known to activate two major neuroendocrine stress-response systems: the sympathetic nervous system (SNS) and the hypothalamic-pituitary-adrenal (HPA) axis (Miller and O'Callaghan, 2002). Activation of the SNS leads to increased release of catecholamines (e.g., norepinephrine), while HPA axis activation results in the secretion of glucocorticoids (e.g., cortisol). These neuroendocrine mediators exert profound modulatory effects on immune cell function (Globig et al., 2023; Müller and Quinkler, 2018). Notably, myeloid cells, including monocytes, macrophages, and granulocytes, are key effector cells of the innate immune system and play a central role in orchestrating inflammatory responses. Crucially, myeloid cells express a rich repertoire of adrenergic and glucocorticoid receptors, rendering them direct targets for stress-related neuroendocrine signals (Walsh et al., 2021). A growing body of evidence indicates that chronic stress, through these neuroendocrine pathways, can directly influence the differentiation, trafficking, and activation state of myeloid cells, skewing their phenotype towards a pro-inflammatory profile and enhancing their capacity to produce and release inflammatory mediators (Walsh et al., 2021; Vasamsetti et al., 2018). Such stress-induced immune alterations, characterized by myeloid cell dysregulation, have been identified as significant drivers in the development and progression of various chronic inflammatory diseases (Grigoriou et al., 2020; Powell et al., 2013; Wohleb et al., 2013).

Based on this, we hypothesized that MD patients experience heightened perceived stress, leading to sustained activation of the SNS and HPA axis. This, in turn, results in elevated plasma catecholamine levels and altered glucocorticoid receptor sensitivity on myeloid cells. These neuroendocrine changes collectively reprogram circulating myeloid cells towards a pro-inflammatory phenotype, characterized by increased expression of pro-inflammatory genes and enhanced release of inflammatory cytokines into the systemic circulation. We further posited that this stress-related, myeloid-mediated systemic inflammation contributes to the deterioration of audio-vestibular function in MD, thereby playing a pivotal role in disease progression.

This study aimed to investigate whether MD patients exhibit elevated perceived stress and to explore its association with neuroendocrine markers (e.g., catecholamine levels, myeloid glucocorticoid receptor expression) and the inflammatory state of myeloid cells. We further sought to assess the correlation between serum inflammatory cytokine levels and the deterioration of audio-vestibular function in MD patients through cross-sectional and follow-up cohort analyses. Finally, in vitro experiments were conducted to elucidate the specific regulatory mechanisms of catecholamine and cortisol on the pro-inflammatory transcriptional program of myeloid cells, thereby aiming to connect the “stress-neuroendocrine-myeloid inflammation-inner ear function” axis in the progression of MD.

## 2 Methods

### 2.1 Participants

**Fig. 1:**
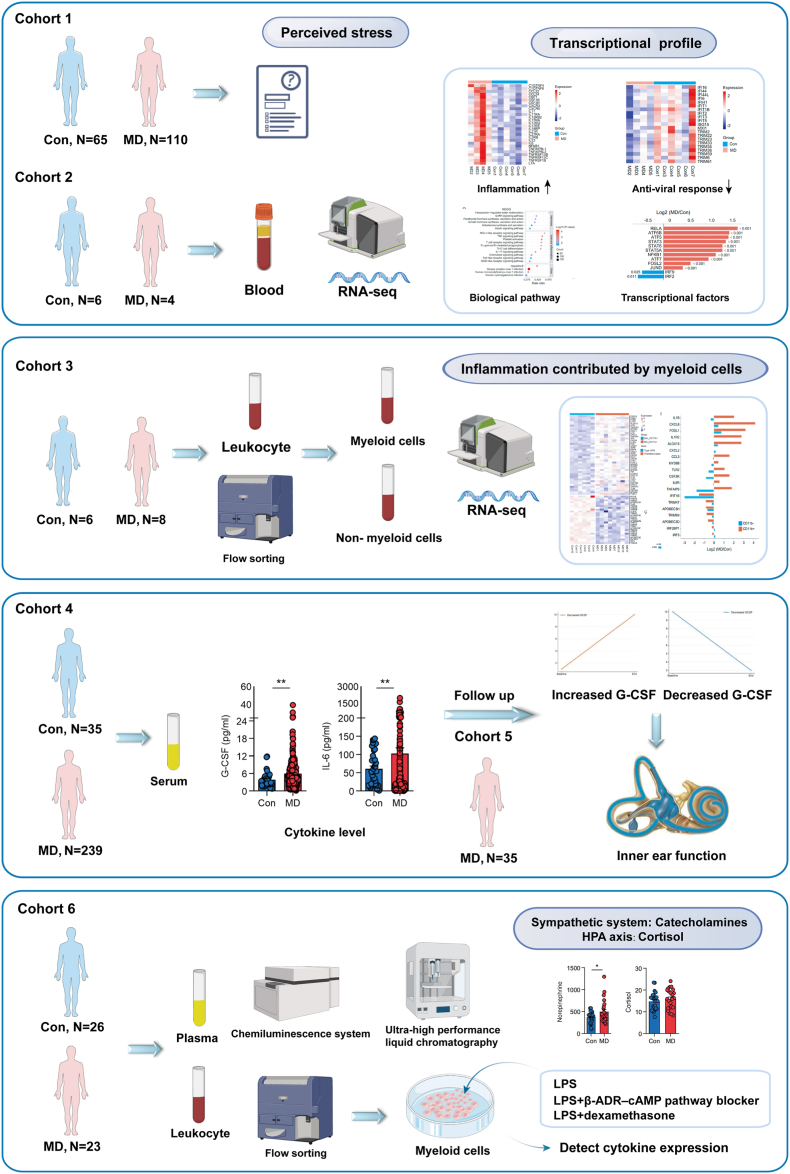
Schematic depicting study design.

This multi-cohort study was conducted at the Vertigo disease Center of Shandong Provincial ENT Hospital, Shandong University. A total of 384 patients with MD and 138 healthy controls (HCs) were enrolled across six distinct cohorts (Fig. 1). Participants were prospectively and consecutively recruited between January 2023 and March 2024. Patients were recruited during their outpatient visits or hospitalizations, while HCs were volunteers recruited from the local community and hospital staff via advertisements. Participants were assigned to specific cohorts based on the study's needs at the time of recruitment and their informed consent for specific procedures (e.g., questionnaires, standard blood draws, or larger volume blood draws for cell sorting). The demographic and clinical characteristics for each cohort are detailed in Supplementary Table S1–S5.

### 2.2 Inclusion and exclusion criteria

All patients were diagnosed with unilateral definite MD according to the 2015 diagnostic criteria of the Bárány Society (Lopez-et al., 2015). The HC group consisted of volunteers without a history of hearing loss, vertigo, or migraine. Comprehensive exclusion criteria were applied to all participants to minimize confounding factors. Individuals were excluded if they had: 1) A known history of systemic autoimmune diseases, autoinflammatory diseases, or significant allergies requiring ongoing treatment. 2) Active or recent infections (e.g., fever, influenza) within the two weeks prior to enrollment. 3) A history of cancer, recent major surgery, or significant trauma. 4)Use of systemic corticosteroids, immunosuppressants, or other immunomodulatory drugs within the three months prior to enrollment. 5) Other otological, neurological, or systemic diseases that could cause vertigo or hearing loss.

All MD patients had complete medical records and underwent a series of tests (auditory and vestibular examinations, high-resolution computed tomography, and Gadolinium-enhanced magnetic resonance imaging) to exclude those with cerebellopontine angle tumors or other intra-cranial space-occupying lesions and to evaluate endolymphatic hydrops.

### 2.3 Cohort descriptions

Cohort 1 was established for a perceived stress assessment and included 110 MD patients (mean age 49.1 ± 14.9 years; 57.3% female) and 65 HCs (mean age 47.7 ± 11.6 years; 43.1% female) (Supplementary Table 1).

Cohort 2 was used for a preliminary exploration of inflammatory transcriptional characteristics. It comprised 4 MD patients (mean age 40.3 ± 13.7 years; 75.0% female) and 6 HCs (mean age 39.7 ± 8.3 years; 33.3% female), from whom peripheral blood was sampled for RNA sequencing and whole-blood validation (Supplementary Table 2).

Cohort 3 was designed to identify the primary cellular origin of inflammation. It included 8 MD patients (mean age 53.8 ± 8.2 years; 75.0% female) and 6 HCs (mean age 54.8 ± 10.3 years; 66.7% female), whose peripheral blood was used for RNA sequencing of sorted myeloid (CD11b+) and non-myeloid (CD11b-) cells (Supplementary Table 3). Subjective stress in cohort 3 was also assessed using the PSS-10 scale.

Cohort 4 was used for the measurement of serum myeloid-derived cytokines and included 239 MD patients (mean age 52.4 ± 12.8 years; 57.7% female) and 35 HCs (mean age 48.6 ± 13.1 years; 51.4% female) (Supplementary Table 4).

Cohort 5 was a follow-up group to correlate cytokine dynamics with clinical progression. It consisted of 42 patients from Cohort 5 who provided an additional blood sample during their follow-up period. The characteristics of these participants are summarized in Table 2.

**Table 1:** Clinical features of patients with Menière's disease in cohort 4 with low and high basal levels of G-CSF.

Characteristic	Mean (SD)			
	MD with low G-CSF (n = 66)	MD with high G-CSF (n = 120)	Statistic	*P* value
Age,Years	52.2 (12.7)	52.3 (15.6)	U = 3908.5	0.883
Female, No. (%)	34 (51.5)	72 (60.0)	χ2 = 1.251	0.263
Left, No. (%)	36 (54.5)	74 (61.7)	χ2 = 0.894	0.345
Age of Oneset (SD)	46.9 (14.5)	47.1 (13.7)	U = 3943.5	0.963
Duration (min), mean (SD)	285.7 (463.8)	301.3 (711.6)	U = 3876	0.809
Migraine, No. (%)	15 (22.7)	26 (21.7)	χ2 = 0.028	0.867
Autoimmune disease, No. (%)	1 (1.5)	2 (1.7)	χ2 = 0.000	1
Allergy	10 (15.2)	10 (8.3)	χ2 = 2.063	0.151
Hypertension, No. (%)	20 (30.3)	26 (21.7)	χ2 = 1.706	0.191
Coronary Heart Disease, No. (%)	2 (3.0)	7 (5.8)	χ2 = 0.245	0.62
Diabetes, No. (%)	5 (7.6)	9 (7.5)	χ2 = 0.000	1
Smoking, No. (%)	7 (10.6)	12 (10.0)	χ2 = 0.017	0.896
PTA, dB	53.0 (18.5)	58.5 (22.2)	U = 3266.5	**0.048**
SDS (%)	56.8 (25.9)	55.3 (29.2)	U = 3602.5	0.967
UW (%)	38.8 (22.2)	36.9 (23.8)	U = 3811.5	0.672
vHIT VOR gain	0.82 (0.22)	0.81 (0.24)	U = 3906.5	0.879
cVEMP abnormal, No. (%)	39 (59.1)	88 (73.3)	χ2 = 3.988	**0.046**
oVEMP abnormal, No. (%)	39 (59.1)	97 (80.8)	χ2 = 10.241	**0.001**
Grading of endolymphatic hydrops, No. (%)				
Normal	8 (12.1)	14 (11.9)	χ2 = 0.409	0.815
Mild	16 (24.2)	24 (20.3)		
Severe	42 (63.6)	80 (67.8)		

Data are shown as mean ± SD. PTA, pure tone average of 0.5, 1 and 2 k Hz; SDS, speech discrimination score; UW, unilateral weakness; vHIT, video head impulse test; cVEMP, vestibular evoked myogenic potential; oVEMP, ocular vestibular evoked myogenic potential. NA, not applicable.

**Table 2:** Clinical features of patients with Menière's disease in cohort 5 with elevated and decreased G-CSF.

Characteristic		MD with elevated G-CSF (n = 20)					MD with decreased G-CSF (n = 15)			
	No.	Baseline	Follow-up	Statistic	*P* value	No.	Baseline	Follow-up	Statistic	*P* value
Age,Years, mean (SD)	20	46.6 (10.5)	NA	NA	NA	15	54.0 (11.3)	NA	NA	NA
Female, No. (%)	20	11 (55.0)	NA	NA	NA	15	7 (46.7)	NA	NA	NA
Left, No. (%)	20	9 (45.0)	NA	NA	NA	15	8 (53.3)	NA	NA	NA
Age of Oneset (y), mean (SD)	20	44.9 (10.8)	NA	NA	NA	15	50.6 (12.7)	NA	NA	NA
Duration (min), mean (SD)	20	245.5 (321.1)	NA	NA	NA	15	312.0 (422.6)	NA	NA	NA
Follow-up time (w), mean (SD)	20	13.0 (12.9)	NA	NA	NA	15	14.1 (11.6)	NA	NA	NA
Migraine, No. (%)	20	5 (25.0)	NA	NA	NA	15	1 (6.7)	NA	NA	NA
Immune disease, No. (%)	20	5 (25.0)	NA	NA	NA	15	1 (6.7)	NA	NA	NA
Allergy, No. (%)	20	4 (20.0)	NA	NA	NA	15	2 (13.3)	NA	NA	NA
Hypertension, No. (%)	20	2 (10.0)	NA	NA	NA	15	4 (26.7)	NA	NA	NA
Coronary Heart Disease, No. (%)	20	0 (0)	NA	NA	NA	15	4 (26.7)	NA	NA	NA
Diabetes, No. (%)	20	0 (0)	NA	NA	NA	15	2 (13.3)	NA	NA	NA
Smoking, No. (%)	20	2 (10.0)	NA	NA	NA	15	0 (0)	NA	NA	NA
G-CSF	20	4.5(8.4)	29.1 (98.4)	Z = −3.920	**0**	15	5.7 (3.9)	2.2 (2.3)	t = 4.592	**0**
IL-6	20	56.8 (90.0)	77.3 (167.4)	Z = −0.411	0.701	15	247.3 (672.9)	22.9 (46.2)	Z = −1.477	0.151
TNF-a	20	3.6 (10.4)	74.5 (306.7)	Z = −3.920	**0**	15	4.1 (6.7)	1.6 (1.3)	t = 1.641	0.123
PTA, dB	20	43.0 (22.0)	46.9 (22.4)	Z = −0.691	0.504	14	36.4 (18.5)	44.8 (27.2)	t = −1.052	0.312
SDS (%)	15	62.4 (29.9)	63.5 (31.1)	Z = −0.314	0.768	11	68.6 (20.7)	62.5 (23.9)	t = 1.060	0.314
UW (%)	9	29.8 (14.8)	51.7 (24.5)	t = −3.236	**0.012**	8	23.9 (18.5)	48.6 (27.3)	t = −2.015	0.084
vHIT VOR gain	12	0.8 (0.2)	0.8 (0.2)	Z = −0.236	0.837	8	0.9 (0.1)	0.7 (0.3)	t = 1.569	0.161
cVEMP abnormal, No. (%)	14	5 (35.7)	12 (85.7)	χ2 = 4.000	**0.039**	13	7 (53.8)	9 (69.2)	χ2 = 0.250	0.625
oVEMP abnormal, No. (%)	14	7 (50.0)	6 (42.9)	χ2 = 0.000	1	13	11 (84.6)	12 (92.3)	χ2 = 0.000	1
Grading of endolymphatic hydrops, No. (%)										
Normal	20	5 (25.0)	NA	NA	NA	15	3 (20.0)	NA	NA	NA
Mild	20	6 (30.0)	NA			15	6 (40.0)	NA		
Severe	20	9 (45.0)	NA			15	6 (40.0)	NA		

Data are shown as mean ± SD. PTA, pure tone average of 0.5, 1 and 2 k Hz; SDS, speech discrimination score; UW, unilateral weakness; vHIT, video head impulse test; cVEMP, vestibular evoked myogenic potential; oVEMP, ocular vestibular evoked myogenic potential. NA, not applicable.

Cohort 6 was used to analyze catecholamine and cortisol signal pathways. This cohort consisted of 23 MD patients (mean age 56.3 ± 10.1 years; 52.2% female) and 26 HCs (mean age 56.8 ± 6.5 years; 34.6% female), whose peripheral blood was used for flow cytometry and in vitro intervention assays (Supplementary Table 5).

### 2.4 Stratification of patient groups for analysis

To investigate the association between G-CSF and clinical outcomes, MD patients were stratified for two separate analyses. First, for the cross-sectional analysis of disease severity, MD patients (from Cohort 4) were dichotomized based on a G-CSF threshold derived from the healthy control group. This threshold was defined as the mean plus two standard errors (mean + 2SE) of serum G-CSF levels in the HCs. Patients with G-CSF levels exceeding this value were assigned to the high G-CSF group (n = 120). Those with G-CSF levels at or below this threshold were assigned to the low G-CSF group (n = 66).

Second, patients from the follow-up cohort (n = 42) were categorized based on the percentage change in their serum G-CSF levels between the baseline and follow-up assessments. Patients exhibiting a ≥25% increase in G-CSF level were classified as the increased G-CSF group (n = 20), representing a state of worsening myeloid-derived inflammation. Conversely, patients showing a ≥25% decrease were classified as the decreased G-CSF group (n = 15), representing a state of improving inflammation. Patients with G-CSF level changes falling between these two cutoffs (i.e., <25% increase and <25% decrease, n = 7) were considered to have stable levels and were excluded from this specific comparative analysis.

### 2.5 Blood sample collection and processing

For all experiments, venous blood samples were collected from participants between 07:00 and 09:00 a.m. after an overnight fast to minimize diurnal variations. The type of collection tube and processing method varied according to the downstream application.

For Serum Analysis (Cytokines): Venous blood (5 mL) was collected into pro-coagulation tubes (Improved Medical, Guangdong, China). Samples were allowed to clot for 30 min at room temperature before being centrifuged at 2000×*g* for 15 min at 4 °C. The resulting serum was were measured using a panel kit and analyzed via FCM.

For Plasma Analysis (Catecholamines and Cortisol): Venous blood (4 mL) was collected into tubes containing EDTA as an anticoagulant. Samples were immediately placed on ice and centrifuged at 1000×*g* for 10 min at 4 °C within 30 min of collection. The plasma supernatant was carefully collected, aliquoted, and stored at −80 °C.

For Cellular Assays (Myeloid Cell Isolation): Venous blood (8-10 mL) was collected into EDTA-anticoagulated tubes. Isolation of peripheral blood mononuclear cells (PBMCs) and subsequent cell sorting were performed within 4 h of blood collection.

For Whole Blood RNA Stabilization: For the systemic transcriptomic analysis in Cohort 2, whole blood (2.5 mL) was collected directly into PAXgene Blood RNA Tubes (QIAGEN, Hilden, Germany) according to the manufacturer's instructions to ensure immediate stabilization of the intracellular RNA profile. Samples were stored at −80 °C until RNA extraction.

### 2.6 Measurement of perceived stress

Perceived Stress Scale-10 (PSS-10) (Cohen et al., 1983) was used to assess how individuals perceive situations in their lives as being stressful. This scale measures the extent to which individuals feel that their lives are unpredictable, uncontrollable, and overloaded within the past month. The questions in the PSS-10 are quite general in nature and it is relatively free of specific content. The questionnaire includes six negative items (1, 2, 3, 6, 9, and 10) and four positive items (4, 5, 7, and 8) for participants to respond to using a five-point rating scale ranging from 0 (never) to 4 (very often). The total score ranges from 0 to 40, with higher scores indicating higher levels of perceived stress. The Chinese version of the PSS-10 has demonstrated good reliability and validity (Sun et al., 2019; Jiang et al., 2023).

### 2.7 mRNA extraction and quantitative real-time PCR (qRT-PCR)

Total RNA was extracted from peripheral blood mononuclear cells (PBMCs) using the RNeasy Mini QIAcube Kit (QIAGEN, Hilden, Germany). The relative expression of the target genes was measured via qRT-PCR using an Eppendorf AG 22331 PCR machine (Hamburg, Germany) according to the manufacturer's instructions. Briefly, qRT-PCR was performed using SYBR Green™ Premix EX Taq™ (RR42LR; Takara Biotechnology, Shiga, Japan). The housekeeping gene *GAPDH* was used to normalize mRNA levels among different samples. Fold-change (FC) differences in mRNA expression were determined using the comparative cycle threshold method (Zhang et al., 2020). The primers used are listed in Additional file 6: Table S6.

### 2.8 FCM analysis of serum cytokines

Venous blood (5 mL) was collected into pro-coagulation tubes (Improved Medical, Guangdong, China) and centrifuged at 2000×*g* for 20 min to separate the serum. The serum levels of IL-6, TNF-α, and G-CSF were measured using a panel kit (AimPlex Biosciences Inc., Pomona, CA, USA) and analyzed via FCM (Beckman Coulter Navios-model 3; Beckman Coulter Inc., Brea, CA, USA) following the manufacturer's instructions. The specifications for FCM analysis are shown in Additional file 7: Table S7. The intra- and inter-assay variability of serum cytokine measurements were CV < 10% and CV < 20%, respectively. Following this, 45 μL of serum and an equal volume of beads were mixed and incubated for 1 h. After incubation, 0.1 mL of wash buffer was added, and the samples were centrifuged for 5 min. The samples were first incubated with biotin-conjugated antibodies for 30 min and then with streptavidin-conjugated antibodies for 20 min. Finally, the read buffer was added. Data were evaluated using the FCAP Array™ software (version 3.0; BD Biosciences, San Jose, CA, USA).

### 2.9 Detection of catecholamines and cortisol

Venous blood (4 mL) was collected from patients on an empty stomach in the supine position between 07:00 and 08:00 a.m. Plasma catecholamines were measured using ultra-high performance liquid chromatography (Waters TQ-S), and plasma cortisol levels were measured using a chemiluminescence system (Cortisol CLIA Microparticles; Autobio, Beijing, China) following the manufacturer's instructions.

### 2.10 Myeloid cell isolation

Venous blood (8.5 mL) was collected into tubes containing EDTA (Improve Medical), mixed with an equal volume of saline water, added to a density gradient centrifuge solution (GE Ficoll-Paque Plus; GE Healthcare Bio-Sciences, Pittsburgh, PA, USA), and centrifuged at 1500×*g* for 30 min. After centrifugation, the intermediate layer of white blood cells was removed and washed with saline. Myeloid cells were isolated via FACS using a flow cytometer (BD FACS Aria III 4L17C). Standard forward scatter width versus area criteria were used to discard doublets and capture singlets. CD11b^+^ gates (CD11b APC and 7AAD) were used to sort myeloid cells. The sorted samples were 98% pure. The collected cells were immediately used for co-culture experiments or cryopreserved in liquid nitrogen for RNA sequencing.

### 2.11 Stimulation of myeloid cells

The β-ADR blocker, propranolol (100 μmol/L; Sigma-Aldrich, St. Louis, MO, USA); β1-ADR blocker, atenolol (1 μmol/L; Sigma-Aldrich); β2-ADR blocker, ICI-118,551 (1 μmol/L; Sigma-Aldrich); adenylate cyclase inhibitor, ddA (0.1 μmol/L; Sigma-Aldrich); and dexamethasone (10 ng/mL; CISEN, Jining, China) were added to myeloid cells within the first hour. The cells were stimulated with *Escherichia coli* lipopolysaccharide (10 ng/mL; Sigma-Aldrich) for 24 h. All experiments were performed in duplicate.

### 2.12 RNA preparation, library construction, and sequencing

Whole blood collected in PAXgene tubes, CD11b^+^ myeloid cells, and CD11b^–^ immune cells were used for total RNA extraction using the PAXgene Blood RNA Kit (QIAGEN) or TRIzol reagent (Invitrogen) according to the manufacturer's instructions. Double-stranded cDNA was synthesized from the extracted mRNA. The double-stranded cDNA was end-repaired and adenylated, and the product was amplified through PCR. After denaturation, a single chain ring was obtained. The final library was prepared by digesting linear DNA molecules that were not cyclized. An Agilent 2100 Bioanalyzer (Agilent Technologies, Savage, MD, USA) was used to measure fragment size and concentration. A single-stranded circular DNA molecule was replicated using rolling rings to form a DNA nanosphere containing >200 copies. DNA nanospheres were added to the mesh holes on the chip using high-density DNA nanochip technology. Combined probes and anchored polymerization were used to obtain a 50/100/150-bp read length.

### 2.13 Bioinformatics analysis

Differential expression tests were performed using the DESeq R package (R Foundation for Statistical Computing, Vienna, Austria). The main goal of this study was to identify differentially expressed genes (DEGs). The protein functions of the identified DEGs were analyzed using Gene Ontology (GO) terms (InterProScan [version 5.14-53.0]) in the cellular component, molecular function, and biological process categories. The Kyoto Encyclopedia of Genes and Genomes (KEGG) database (KAAS [version 2.0]; KEGG Mapper [version 2.5]) was used to annotate protein pathways. For the functional enrichment of GO terms and KEGG pathways, a two-tailed Fisher's exact test was used, and a corrected *P*-value <0.05 was considered statistically significant. The heatmap, bubble enrichment diagram, and bar chart were plotted by the Bioinformatics Online Analysis Platform (website: https://www.bioinformatics.com.cn), an online platform for data analysis and visualization, whose functions are coded with Python and/or R^24^.

### 2.14 Statistical analysis

Prior to analysis, quantitative data were assessed for normal distribution using the Shapiro-Wilk test. For comparisons between two independent groups (e.g., MD vs. HCs), Student's unpaired *t*-test was used for normally distributed data, while the non-parametric Mann-Whitney *U* test was used for data that were not normally distributed. For paired comparisons within the follow-up cohort, the Wilcoxon signed-rank test was used. Categorical data are presented as numbers and percentages and were compared using the chi-square test or Fisher's exact test where appropriate. Correlation analysis was performed using Spearman's rank correlation coefficient (r) to assess monotonic relationships between variables. Quantitative data are presented as mean ± standard deviation (SD) unless otherwise specified. Statistical analyses were performed using SPSS (version 21.0; SPSS Inc., Chicago, IL, USA). A two-tailed P-value <0.05 was considered statistically significant.

## 3 Results

### 3.1 Patients with MD exhibit increased perceived stress and a systemic pro-inflammatory transcriptional signature

To assess the level of psychological stress associated with MD, PSS-10 scores from Cohort 1 were compared across the MD and healthy groups. Patients with MD scored significantly higher than the control group (p < 0.05), indicating an increased global level of perceived stress in individuals with MD (Table S1).

**Fig. 2:**
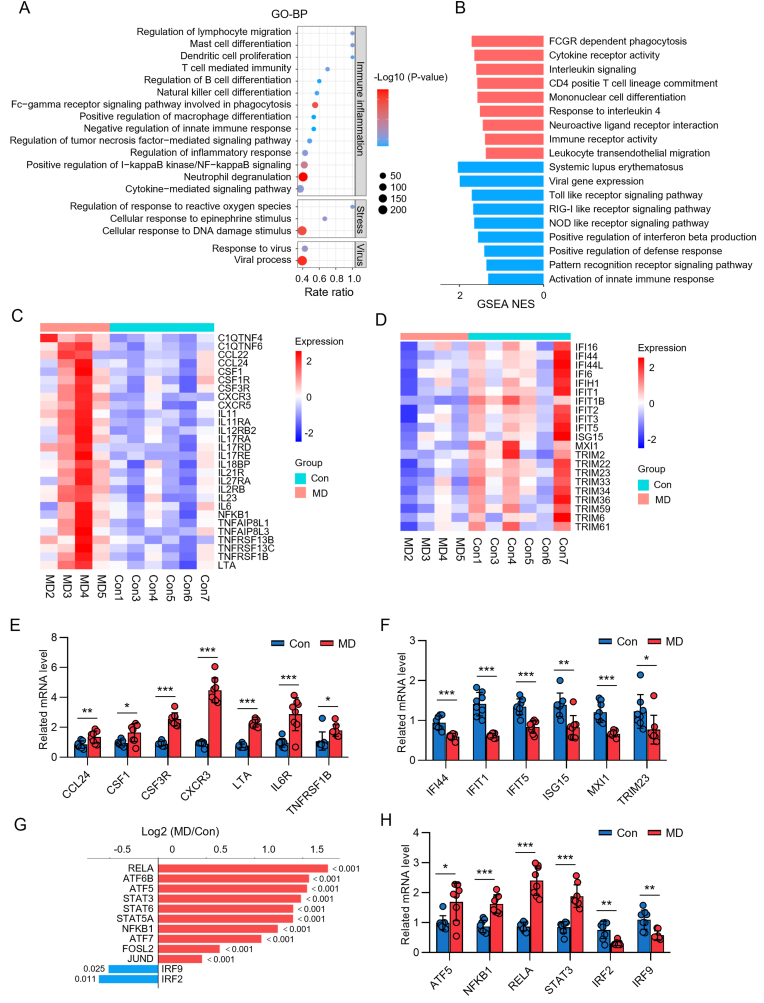
Transcriptional Signature in MD from cohort 2. **A** Bubble diagrams representing the enrichment analysis of the DEGs enriched in the biological process category. **B** Gene set enrichment analysis. **C** Heatmap of pro-inflammatory differentially expressed genes (DEGs). **D** Heatmap of anti-viral type I IFN pathway related DEGs. **E** qRT-PCR analysis of CCL24, CSF1, CSF3R, CXCR3, LTA, IL-6R, and TNFRSF1B in PBMCs. **F** qRT-PCR analysis of IFI44, IFIT1, IFIT5, ISG15, MX1, and TRIM23 in PBMCs. **G** Bi-directional bar plot of directionality (blue, down-regulated; red, up-regulated). **H** qRT-PCR analysis of ATF5, NFKB1, RELA, STAT3, IRF2, and IRF9 in PBMCs. BP, biological process; Con, control; NES, normalized enrichment score. ∗*P* < 0.05; ∗∗*P* < 0.01; ∗∗∗*P* < 0.001. (For interpretation of the references to colour in this figure legend, the reader is referred to the Web version of this article.)

To define the systemic transcriptional alterations in MD, RNA sequencing was performed on peripheral blood samples from four patients with MD and six age- and sex-matched healthy volunteers in cohort 2. The two groups were significantly separated (Fig. S1A). A total of 5725 DEGs (2795 up-regulated and 2930 down-regulated) were screened according to the criteria of | log2 FC | ≥ 1 and Q < 0.05 (Fig. S1B–D). Initial functional analysis of these DEGs pointed towards enrichment of immune, stress, and innate anti-viral response pathways (Fig. 2A; Fig. S1E).

To further resolve this immunological signature, Gene Set Enrichment Analysis (GSEA) of peripheral blood transcripts revealed a significant upregulation of pro-inflammatory pathways (Mast cell differentiation, Dendritic cell proliferation, Innate immune response, Regulation of I-kappaB kinase/NF-kappaB signaling, *etc.*) in patients with MD. Concurrently, we observed a suppression of innate anti-viral signaling cascades, including the Toll-like, RIG-I-like, and NOD-like receptor pathways, relative to healthy controls (Fig. 2B). This transcriptional imbalance was characterized by increased expression of pro-inflammatory genes alongside decreased expression of genes integral to the type I interferon (IFN) anti-viral response (Fig. 2C–F; Fig. S1F–G).

At the regulatory level, this dichotomy was mirrored by the differential expression of key transcription factors. Those driving pro-inflammatory programs, such as the NF-κB pathway, were upregulated, whereas transcription factors governing the type I IFN response were downregulated (Fig. 2G–H).

Together, these data define a distinct immunological signature in MD, where the innate immune system is skewed towards a pro-inflammatory state at the expense of anti-viral capacity. This profile is a known hallmark of cellular reprogramming driven by chronic psychosocial adversity (Cole, 2019), thus providing a molecular basis for the link between perceived stress and immune dysregulation in patients with MD.

### 3.2 Myeloid cells are Key Contributors to the pro-inflammatory signature in MD

**Fig. 3:**
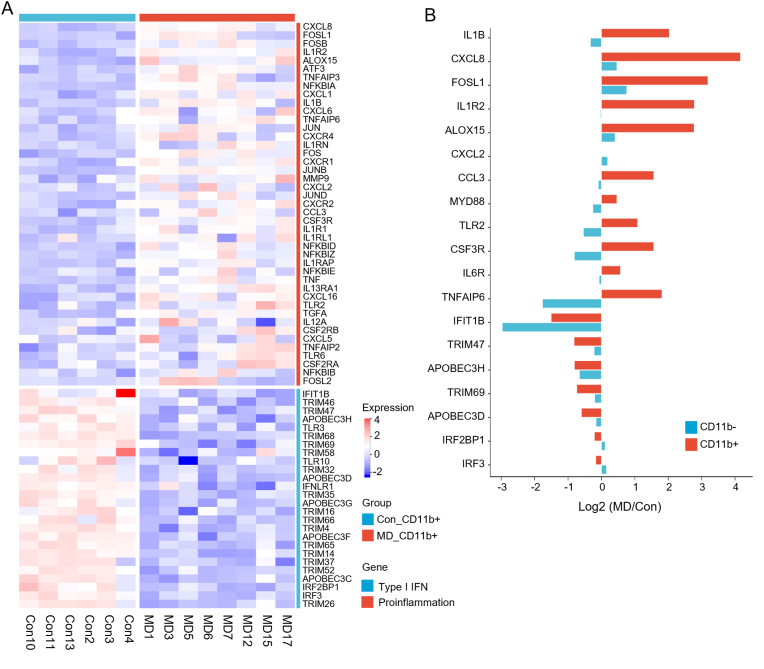
Myeloid Cells as Key Contributors to the inflammatory Signature in MD. **A** Heatmap of pro-inflammatory differentially expressed genes (DEGs) and DEGs related to the type I IFN pathway in myeloid cells. **B** Bi-directional bar plot of directionality (CD11b^+^ and CD11b^–^ cells) (blue, down-regulated; red, up-regulated). The data were derived from Cohort 3. (For interpretation of the references to colour in this figure legend, the reader is referred to the Web version of this article.)

To identify the primary cellular contributors to the observed systemic pro-inflammatory transcriptional signature, we next focused on myeloid cells, key effector cells of the innate immune system. CD11b + myeloid cells were isolated by fluorescence-activated cell sorting (FACS) from peripheral blood of patients with MD and healthy volunteers in Cohort 3 for RNA sequencing (Fig. S2A). Analysis of the myeloid cell transcriptome revealed 805 DEGs (542 up-regulated and 263 down-regulated) in MD patients compared to controls, using the criteria of |log2 FC| ≥ 1 and Q < 0.05 (Fig. S2B–D). KEGG pathway analysis of these DEGs in myeloid cells showed significant enrichment in immune-related pathways, innate anti-viral immune responses, and hematopoietic cell lineage pathways (Fig. S2E). Crucially, direct comparison of gene expression within these isolated myeloid cells demonstrated that those from MD patients exhibited significantly increased expression of pro-inflammatory genes and concomitantly decreased expression of anti-viral genes relative to myeloid cells from healthy controls (Fig. 3A).

To further confirm the central role of myeloid cells in shaping the systemic transcriptional changes observed in MD, we compared the fold changes of representative pro-inflammatory and anti-viral genes in CD11b + myeloid cell versus those in PBMCs depleted of myeloid cells (CD11b-). The up-regulation of key pro-inflammatory genes and the down-regulation of key anti-viral genes, prominent in the CD11b + myeloid cell of MD patients, were substantially reduced or even reversed when myeloid cells were experimentally removed (Fig. 3B). This indicates that the distinct pro-inflammatory and anti-viral suppressed transcriptional signature observed in the peripheral blood of MD patients is predominantly stemming from alterations within the myeloid cell compartment.

Next, we explored the relationship between the transcriptional levels of myeloid pro-inflammatory genes and stress levels in Cohort 3 (Fig. S2F). Correlation analysis revealed that most pro-inflammatory genes were significantly positively correlated with PSS-10 scores, including chemokines involved in neutrophil chemotaxis and activation (such as CXCL1, CXCL2, and CXCL8) and their receptors (CXCR1, CXCR2).

### 3.3 Myeloid-derived inflammatory cytokines correlate with MD severity and progression

**Fig. 4:**
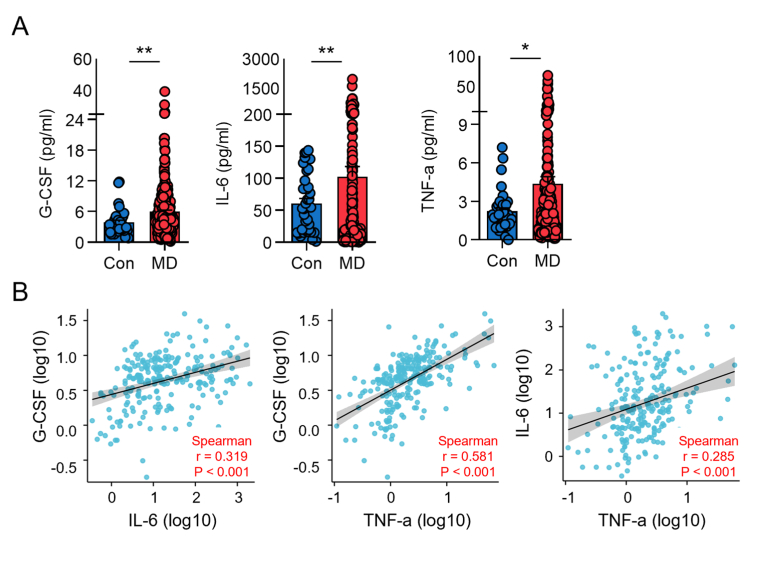
Increased levels of myeloid-derived cytokines in patients with MD from cohort 4. **A.** Serum levels of G-CSF, IL-6, and TNF-α determined by FCM. **B.** Spearman correlation analysis between G-CSF, IL-6, and TNF-α. Con, control. ∗*P* < 0.05; ∗∗*P* < 0.01.

We next investigated how the pro-inflammatory functions of myeloid cells affect the clinical phenotypes of MD. We selected and tested G-CSF, IL-6, and TNF-α to assess myeloid cell pro-inflammatory function for three reasons. First, all three are classic myeloid-derived cytokines that reflect the functional state of this cell population. Second, previous studies have reported that G-CSF is elevated in MD patients and correlates with disease severity (Zou et al., 2022). Finally, IL-6 and TNF-α are well-established markers induced by psychosocial stress and sympathetic nervous system activation, which aligns perfectly with our study's central hypothesis (Schiller et al., 2021). Therefore, we first investigated the serum levels of these three key myeloid-derived cytokines between MD and HC group in Cohort 4. The results showed that the serum levels of G-CSF, IL-6, and TNF-α in patients with MD were significantly higher than those in healthy volunteers (Fig. 4A). Correlation analyses further revealed that G-CSF levels were positively and significantly correlated with both IL-6 and TNF-α levels, suggesting that G-CSF could serve as a robust indicator of the myeloid inflammatory burden (Fig. 4B). To examine the association between this inflammatory marker and clinical severity, we stratified MD patients into high (n = 120) and low (n = 66) G-CSF groups based on the mean ±2SE of serum G-CSF in the control group. The results showed that patients in the high G-CSF group had poorer hearing and vestibular function (Table 1, Fig. S3), indicating that the magnitude of systemic, myeloid-derived inflammation is associated with the severity of audio-vestibular impairment.

To evaluate the effect of myeloid-derived cytokines on the progression of MD, 42 patients with MD were recruited and followed up (cohort 5). Serum G-CSF levels and indicators of audio-vestibular function were measured at baseline and at the follow-up assessment. Based on a ≥25% change of G-CSF levels at follow-up, patients with MD were divided into increased (*n* = 20) and decreased (*n* = 15) G-CSF groups, representing the group with increased myeloid-derived inflammation and the group with decreased myeloid-derived inflammation at follow-up, respectively (Table 2). Audio-vestibular function deteriorated in the increased G-CSF group but remained stable in the decreased G-CSF group (Table 2). Collectively, these cross-sectional and follow-up findings suggest that myeloid cell-derived systemic inflammation, robustly indicated by G-CSF levels, is not only associated with the severity of MD symptoms but also plays a potential role in predicting disease progression.

### 3.4 Role of catecholamines in myeloid cell proinflammatory priming

**Fig. 5:**
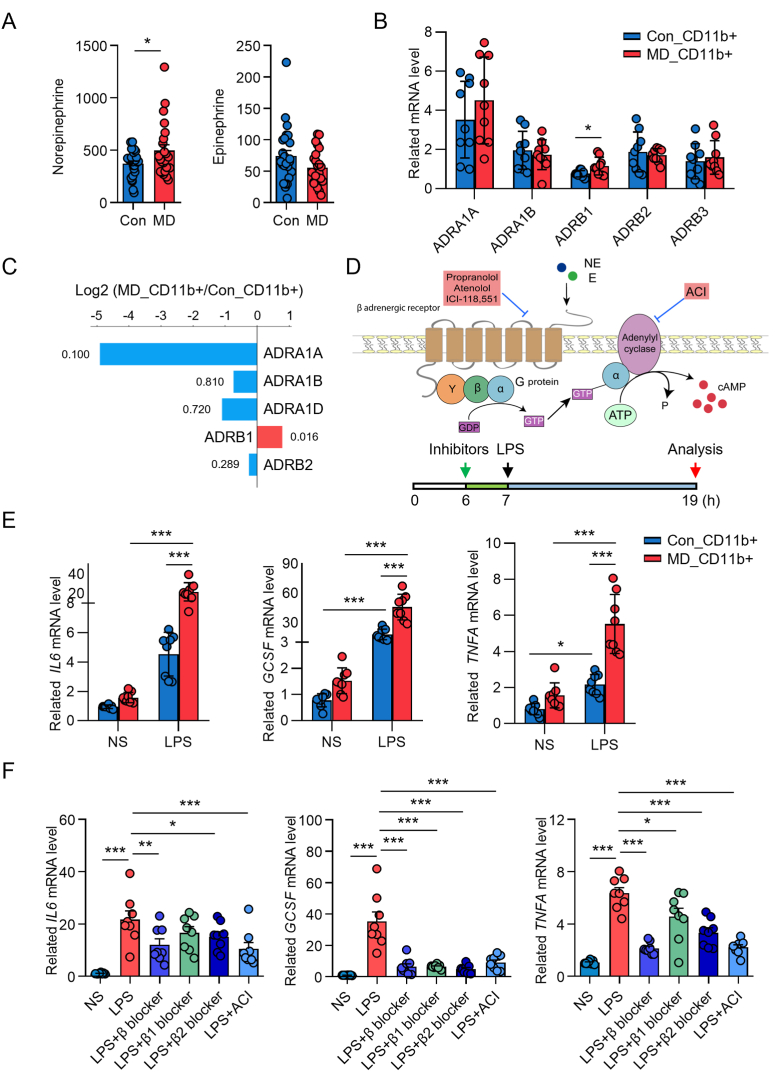
Regulation of the catecholamines in myeloid cells. **A** Plasma norepinephrine and epinephrine levels. **B** qRT-PCR analysis of ADRA1A, ADRA1B, ADRB1, ADRB2, and ADRB3 in CD14^+^ cells. **C** Bi-directional bar plot of directionality (ADR) (blue, down-regulated; red, up-regulated). **D** Flowchart of the experimental scheme. **E** qRT-PCR analysis of IL-6, G-CSF, and TNF-α in CD11b^+^ cells treated with saline or lipopolysaccharide for 24 h. **F** qRT-PCR analysis of IL-6, G-CSF, and TNF-α in CD11b^+^ cells treated with saline, lipopolysaccharide, or inhibitors for 24 h. Con, control. ∗*P* < 0.05; ∗∗*P* < 0.01; ∗∗∗*P* < 0.001. The data were derived from Cohort 6. (For interpretation of the references to colour in this figure legend, the reader is referred to the Web version of this article.)

Given the potential link between psycho-behavioral stressors and immune alterations in MD, we investigated markers of SNS activity. We measured circulating norepinephrine and epinephrine levels and found that serum norepinephrine concentrations were significantly higher in patients with MD than in healthy volunteers in cohort 6 (Fig. 5A). In contrast, epinephrine levels showed no significant difference between the two groups (Fig. 5A). These findings suggest a state of heightened SNS tone in MD patients, primarily reflected by elevated norepinephrine.

To explore whether myeloid cells in MD patients might exhibit altered responsiveness to sympathetic signals, we assessed the expression of adrenergic receptors. RNA sequencing of CD11b + myeloid cells revealed that mRNA levels of the β1-adrenergic receptor (ADRB1) were increased in patients with MD compared to healthy controls (Fig. 5C). This upregulation of ADRB1 mRNA in CD11b + myeloid cells from MD patients was further validated by quantitative PCR (Fig. 5B), suggesting an enhanced potential for catecholaminergic signaling in these cells.

“The β-adrenergic receptor (β-ADR)–cyclic AMP (cAMP) pathway is a key signaling cascade that transduces extracellular catecholamine signals into intracellular responses (Fig. 5D) (Kolmus et al., 2015). To determine the long-term effects of β-ADR–cAMP pathway activation by catecholamines on myeloid cell inflammatory responses, CD11b + myeloid cells from patients with MD and healthy volunteers were stimulated in vitro with lipopolysaccharide (LPS). Following LPS stimulation, myeloid cells from MD patients exhibited significantly higher mRNA levels of the pro-inflammatory cytokines IL-6, G-CSF, and TNF-α compared to those from healthy volunteers, suggesting an exaggerated or ‘primed’ inflammatory response in MD myeloid cells (Fig. 5E).

To dissect the role of the β-ADR–cAMP pathway in this phenomenon, we employed pharmacological inhibitors. Pre-treatment with either a β1-ADR blocker (atenolol) or a β2-ADR blocker (ICI-118551) partially alleviated the LPS-induced cytokine production. Notably, combined β-ADR blockade using the non-specific β-ADR blocker propranolol effectively prevented the increase in cytokine production (Fig. 5F). Furthermore, addition of the adenylyl cyclase inhibitor, ddA, which inhibits cAMP production downstream of β-ADRs, abolished the pro-inflammatory response (Fig. 5F). Although the amplitude of responsiveness to stimulation varied between individuals, the inhibitory effect of β-ADR–cAMP pathway inhibitors on the pro-inflammatory immune response was consistent across samples. These results collectively indicate that sympathetic signaling, acting through the β-ADR–cAMP pathway, contributes to the pro-inflammatory priming of myeloid cells in patients with MD.

### 3.5 Altered glucocorticoid signaling in myeloid cells

**Fig. 6:**
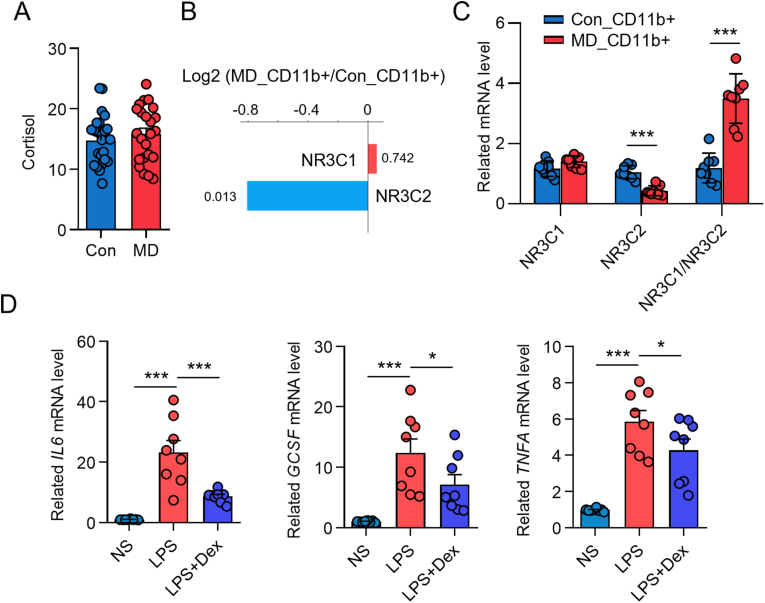
Anti-inflammatory effects of glucocorticoids on myeloid cells. **A** Plasma cortisol levels. **B** Bi-directional bar plot of directionality (NR3C1 and NR3C2) (blue, down-regulated; red, up-regulated). **C** qRT-PCR analysis of NR3C1 and NR3C2 in CD11b^+^ cells. **D** qRT-PCR analysis of IL-6, G-CSF, and TNF-α in CD11b^+^ cells treated with saline, lipopolysaccharide, or dexamethasone for 12 h. Con, control. ∗*P* < 0.05; ∗∗∗*P* < 0.001. The data were derived from Cohort 6. (For interpretation of the references to colour in this figure legend, the reader is referred to the Web version of this article.)

The HPA axis, culminating in cortisol secretion, represents another critical stress-response system with profound immunomodulatory effects. We first examined systemic cortisol levels and found no significant differences in blood cortisol concentrations between patients with MD and healthy volunteers in cohort 6 (Fig. 6A). However, given that cellular responsiveness to glucocorticoids is also determined by receptor expression, we next investigated glucocorticoid receptor (GR) and mineralocorticoid receptor (MR, encoded by NR3C2) expression in myeloid cells. RNA sequencing analysis of CD11b + myeloid cells revealed a significant reduction in NR3C2 (MR) mRNA levels in patients with MD compared to controls, while NR3C1 (GR) mRNA expression showed no significant difference (Fig. 6B). These findings were subsequently confirmed by quantitative PCR (Fig. 6C), indicating an altered glucocorticoid receptor profile specifically characterized by lower MR expression in myeloid cells of MD patients, despite normal circulating cortisol levels.

To assess whether the observed alterations in MR expression impact the ability of myeloid cells from MD patients to respond to glucocorticoid-mediated immunosuppression, we performed in vitro experiments. CD11b + myeloid cells from MD patients were stimulated with LPS in the presence or absence of dexamethasone, a synthetic glucocorticoid. The results demonstrated that dexamethasone significantly inhibited the LPS-induced upregulation of IL-6, G-CSF, and TNF-α mRNA levels in these cells (Fig. 6D). This finding suggests that, despite the reduced expression of NR3C2 (MR), myeloid cells from MD patients retain their capacity to respond to the anti-inflammatory effects of pharmacological doses of glucocorticoids, which may be relevant to the therapeutic efficacy of corticosteroids in MD management.

## 4 Discussion

**Fig. 7:**
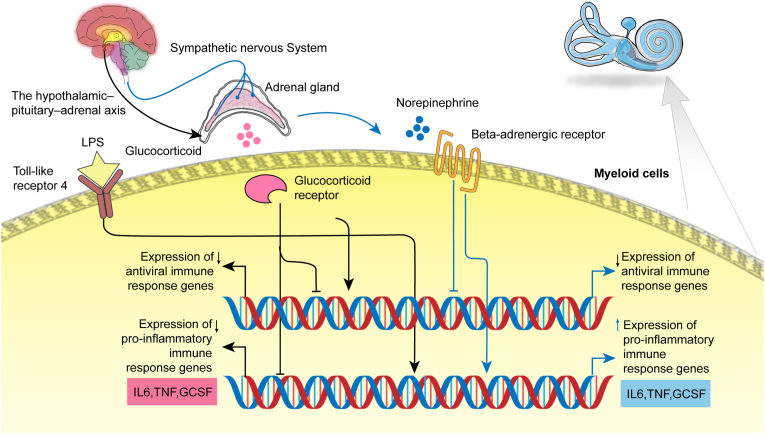
Proposed model of stress-induced neuroimmune modulation of myeloid cells in Ménière's disease. Psychosocial stress activates two major neuroendocrine pathways, which collaboratively shape the immune profile. (1) The Sympathetic Nervous System (SNS) releases norepinephrine, which binds to beta-adrenergic receptors on myeloid cells. This signaling pathway enhances the cellular response to inflammatory stimuli (e.g., LPS binding to Toll-like receptor 4), leading to potentiated expression of pro-inflammatory immune response genes and increased production of cytokines such as IL-6, TNF, and G-CSF, while simultaneously downregulating the expression of antiviral response genes. (2) Concurrently, the hypothalamic-pituitary-adrenal (HPA) axis is activated, causing the adrenal gland to release glucocorticoids. These hormones act via the glucocorticoid receptor to exert a broad immunosuppressive effect, which not only suppresses the pro-inflammatory response but also contributes to the downregulation of antiviral gene expression. In a state of chronic stress, a dysregulation of this balance—potentially characterized by SNS overactivity and/or altered glucocorticoid sensitivity—can lead to a dominant pro-inflammatory phenotype coupled with impaired antiviral immunity, contributing to MD pathology.

The intricate interplay between the nervous and immune systems is a critical component of human diseases (Schiller et al., 2021; Leunig et al., 2025). In MD, the link between psychological stress and disease progression is well-documented but has lacked a clear cellular and molecular basis (Ishii et al., 2022; Andersson et al., 1997; Fujita et al., 2023). Our study addresses this fundamental gap by identifying CD11b + myeloid cells as a key cellular hub connecting these systems. We demonstrate that these cells are reprogrammed towards a pro-inflammatory state via neuroendocrine signaling, specifically through elevated norepinephrine and altered adrenergic receptor expression. Furthermore, we establish that myeloid-derived cytokines, are associated with both the severity and progression of inner ear dysfunction. Collectively, these findings provide a novel point of stress-neuroendocrine-myeloid-inflammation, offering a new mechanistic framework for the pathophysiology of MD (Fig. 7).

Chronic stress exposure typically triggers a cascade of physiological adaptations. Our investigation confirmed that patients with MD report significantly higher levels of perceived stress. RNA sequencing of peripheral blood cells from MD patients revealed a distinct gene expression signature characterized by a significant upregulation of pro-inflammatory genes and a downregulation of genes related to the anti-virus response. This specific down-regulation of the anti-viral response offers a potential molecular mechanism for the proposed susceptibility of MD patients to viral triggers or the reactivation of latent viruses (Kurtzman and Sioshansi, 2023). Cell-type original analyses further indicated that this pro-inflammatory phenotype was predominantly attributable to alterations within CD11b + myeloid cells. This finding suggests that sustained psychosocial stress in MD may be a critical upstream driver for skewing myeloid cells towards a pro-inflammatory state. Indeed, prolonged stress exposure has been shown to reprogram the transcriptional landscape of immune cells, particularly myeloid cells, favoring the expression of pro-inflammatory genes and leading to an immune bias that, while potentially beneficial for acute threats, can be detrimental in chronic conditions. Our finding of a myeloid-driven inflammation converges with recent reports that have established monocytes as a primary source of pro-inflammatory cytokines in MD (Flook et al., 2023; Frejo et al., 2018; Moleon et al., 2021).Building upon this established role, it is crucial to place this finding within the broader context of MD's complex immunopathology. The immune dysregulation in MD is not confined to a single lineage but is a multifaceted process involving coordinated responses across both the myeloid and lymphoid arms. Indeed, previous work has already established the pro-inflammatory involvement of NK and CD4^+^ T cells (Flook et al., 2023; Cruz-Granados et al., 2024). Therefore, our results add a critical dimension to this picture by identifying a specific myeloid-centric signature that likely interacts with these known lymphoid responses.

We observed significantly elevated plasma norepinephrine levels in MD patients, accompanied by an upregulation of β1-adrenergic receptor (ADRB1) mRNA in their myeloid cells. This suggests not only an activated SNS but also potentially heightened sensitivity or exposure of peripheral myeloid cells to catecholamine signaling in MD. Catecholamines, by activating β-adrenergic receptors on myeloid cells, particularly via the β-ADR–cAMP pathway, mediate changes in cellular function. Our in vitro experiments strongly support this: blockade of β-ADRs (non-selective, β1-selective, or β2-selective) or inhibition of downstream adenylyl cyclase significantly attenuated lipopolysaccharide (LPS)-induced production of pro-inflammatory cytokines by myeloid cells. This indicates that chronic sympathetic overactivity in MD may “prime” myeloid cells via the β-ADR–cAMP pathway, leading to an exaggerated pro-inflammatory response upon subsequent immune challenge. The involvement of both β1 and β2 subtypes in this pro-inflammatory response may reflect the complexity of MD pathophysiology, as β-receptor subtype roles can vary across different diseases or models (Ince et al., 2018; Scanzano and Cosentino, 2015). Our data suggest that specific molecular strategies targeting the β-ADR–cAMP pathway, such as β-blockers, could offer novel therapeutic avenues for MD, a concept supported by studies demonstrating the anti-inflammatory potential of β-blockers in other chronic conditions (Haldar et al., 2018; Jachs et al., 2021; Shaashua et al., 2017).

The HPA axis, another critical stress-response system, culminates in the release of cortisol, which possesses broad anti-inflammatory effects and are commonly used in MD treatment, although their precise mechanisms of action remain incompletely understood (Sajjadi and Paparella, 2008). Interestingly, we found no significant difference in plasma cortisol levels between MD patients and healthy controls. However, a deeper analysis of glucocorticoid receptor expression in myeloid cells revealed a significant downregulation of NR3C2 (encoding the mineralocorticoid receptor, which also acts as a low-affinity glucocorticoid receptor) in MD patients, while NR3C1 (encoding the classical glucocorticoid receptor) expression remained unchanged. As NR3C1 primarily mediates the anti-inflammatory effects of glucocorticoids, and NR3C2 may negatively regulate NR3C1 function, an increased NR3C1/NR3C2 expression ratio could imply heightened sensitivity of myeloid cells to the anti-inflammatory effects of glucocorticoids in MD patients. This aligns with the clinical observation that many MD patients respond favorably to glucocorticoid therapy. Our in vitro studies confirmed that dexamethasone effectively suppressed LPS-induced inflammation in myeloid cells. This suggests that even with normal systemic cortisol levels, alterations in glucocorticoid sensitivity at the myeloid cell level could influence their inflammatory status. For MD patients who respond poorly to glucocorticoids, insensitivity or resistance, as observed in some autoimmune or allergic diseases (Barnes and Adcock, 2009), might be considered. The exact regulatory mechanisms underlying this receptor phenotype change in MD myeloid cells remain unclear and could represent a compensatory mechanism or feedback loop in response to inflammation.

The functional consequence of this myeloid cell priming was evident at the systemic level (Zou et al., 2022). Our cross-sectional and follow-up data strongly suggest that this systemic inflammatory milieu, related to inner ear function and MD progression. This raises the critical question of how systemic inflammation translates into localized inner ear pathology. The blood-labyrinth barrier (BLB), a physical barrier segregating the inner ear, may be a key interface. While once viewed as impervious, recent evidence suggests that BLB integrity can be compromised in MD (Bernaerts et al., 2019; Pakdaman et al., 2016). Systemic inflammation is a well-known driver of vascular permeability, and it is plausible that cytokines from the circulation disrupt the BLB. This could allow inflammatory mediators and even non-resident immune cells, such as activated monocytes from the circulation or bone marrow, to infiltrate the inner ear and participate in local immuno-inflammatory responses, as has been shown under other pathological conditions (Hu et al., 2018; Okano et al., 2008). Our previous work reporting sterile inflammation in the MD inner ear due to SGK1 downregulation (Zhang et al., 2023) further supports this concept. This suggests a “two-hit” model, where an inherently susceptible inner ear microenvironment in MD is further damaged by an aggressive systemic inflammatory onslaught driven by stress. In addition, inflammatory cues can alter the hematopoietic stem cell niche and skew hematopoietic output towards the production of more pro-inflammatory myeloid cells, a phenomenon sometimes termed “trained immunity” or emergency myelopoiesis (Anderson et al., 2020). The enrichment of hematopoiesis-related pathways in the differentially expressed genes from our MD cohort lends indirect support to this hypothesis, suggesting that stress-induced inflammation may fundamentally alter the long-term programming of the immune system, although further validation is required.

An important observation is the heterogeneity observed in our immunological data (Fig. 4, Fig. 5): a subset of patients exhibits a particularly robust pro-inflammatory response. We hypothesize that these patients are likely individuals with higher levels of psychosocial stress, or alternatively, that stress-induced myeloid cell priming does not occur uniformly but is most pronounced in a susceptible subgroup of patients. Future studies should concurrently assess psychological, neuroendocrine, and immunological variables within the same individuals to definitively validate this association and explore its clinical implications.

This study has several limitations that should be acknowledged. First, the statistical power of our initial transcriptomic analyses is constrained by the small sample sizes, which were designed for exploratory, hypothesis-generating purposes. The analysis of serum cytokines in Cohort 4 is limited by a sample size imbalance between patients and healthy controls; however, this cohort was primarily designed to assess correlations within the patient group, and non-parametric tests robust to this imbalance were used for group comparisons. Second, a key limitation of this study is that we did not collect direct measurements of psychosocial stress or activation of the HPA axis/SNS and cytokines in the same cohort. Consequently, we were unable to formally verify the mediating role of inflammation. Finally, while this study identifies a key role for the broad CD11b + myeloid compartment, a more granular understanding is needed. Future work should dissect the contributions of specific myeloid subsets (monocytes, macrophages, neutrophils) and explore their functional interplay with the pro-inflammatory lymphoid cells previously implicated in MD.

Our findings provide novel insights into a multi-step pathway through which psychosocial stress may contribute to the progression of MD. This framework suggests that therapeutic strategies aimed at breaking the “stress-neuroendocrine-myeloid inflammation” chain could be beneficial. Effective stress management, through lifestyle modifications (e.g., meditation, Tai Chi) or pharmacological interventions that modulate the stress response (e.g., β-blockers), may help restore immune homeostasis and potentially slow the functional decline in MD patients by mitigating chronic inflammation at its source.

## CRediT authorship contribution statement

**Xiaofei Li:** Writing – review & editing, Writing – original draft, Investigation, Formal analysis, Conceptualization. **Na Zhang:** Writing – review & editing, Writing – original draft, Investigation, Formal analysis, Conceptualization. **Yongdong Song:** Resources. **Tongtong Zhang:** Resources. **Yafeng Lyu:** Resources. **Huirong Jian:** Resources. **Yawei Li:** Resources. **Jing Wang:** Resources. **Wenjuan Li:** Resources. **Yinghui Hu:** Resources. **Zhaomin Fan:** Writing – review & editing, Resources. **Na Li:** Writing – review & editing, Conceptualization. **Daogong Zhang:** Writing – review & editing, Conceptualization. **Haibo Wang:** Writing – review & editing, Conceptualization.

## Ethics approval and consent to participate

The study was performed according to the principles of the Declaration of Helsinki, as revised in 2013, and the guidelines of Shandong Provincial ENT Hospital. The study was approved by the Ethics Committee of Shandong University (approval number: 2023-023-01). Written informed consent was obtained from all participants.

## Availability of data and material

The raw sequence data reported in this article have been deposited in Gene Expression Omnibus (GSE244980).

## Funding

This study was supported by the Shandong Province medicine and health project [grant numbers 202307010309 and 202307010241], National Natural Science Foundation of China [grant numbers 82271172, 82171150, and 82401358]; Major Program of National Natural Science Foundation of China [grant number 82196821]; Natural Science Foundation of Shandong Province [grant number ZR2024QH421]; China Postdoctoral Science Foundation [grant number 2024M761821].

## Declaration of competing interest

The authors declare no competing interests.

## Data Availability

Data will be made available on request.
