# Supplementary material for: A Stress-Neuroendocrine-Myeloid Inflammation Axis Is Associated with the Progression of Ménière's Disease
Source: Brain Behav Immun Health. 2026 Feb 6;52:101193. doi: 10.1016/j.bbih.2026.101193 (PMC12907869; doi:10.1016/j.bbih.2026.101193)
Supplement: Multimedia component 1 [file mmc1.docx]

**Supplementary Appendix**

Supplementary Table 1. Demographical characteristics and perceived stress of cohort 1

| Characteristic | Mean (SD) |  |  |  |
| --- | --- | --- | --- | --- |
|  | MD (n=110) | Controls (n=65) | Statistic | P value |
| Age,Years | 49.1 (14.9) | 47.7 (11.6) | U=3220.000 | 0.431 |
| Female, No. (%) | 63 (57.3) | 28 (43.1) | χ2=3.299 | 0.069 |
| Perceived stress | 16.98 (6.8) | 11.5 (6.3) | U=2041.500 | **0** |

Supplementary Table 2. Demographical characteristics of cohort 2.

| Variables | Controls (n = 6) | MD (n = 4) | Statistic | *P* |  |
| --- | --- | --- | --- | --- | --- |
|  |  |  |  |  |  |
| Age, Years, Mean ± SD | 39.7 ± 8.3 | 40.3 ± 13.7 | t=-0.08 | 0.935 |  |
| Gender, n(%) |  |  | - | 0.524 |  |
| Female | 2 (33.33) | 3 (75.00) |  |  |  |
| Male | 4 (66.67) | 1 (25.00) |  |  |  |
|  |  |  |  |  |  |

|  |  |
| --- | --- |

Supplementary Table 3. Demographical characteristics of cohort 3

| Variables | Control  (n = 6) | MD  (n = 8) | Statistic | *P* |  |
| --- | --- | --- | --- | --- | --- |
|  |  |  |  |  |  |
| Age, Years, Mean ± SD | 54.8 ± 10.3 | 53.8 ± 8.2 | t=0.22 | 0.830 |  |

| Gender, n(%) |  |  | - | 1.000 |  |
| --- | --- | --- | --- | --- | --- |
| Female | 4 (66.67) | 6 (75.00) |  |  |  |

| Male | 2 (33.33) | 2 (25.00) |  |  |  |
| --- | --- | --- | --- | --- | --- |
| PSS-10 score | 11.0（4.5） | 17.9（6.0） | t=2.35 | 0.027 |  |
| The time interval since the last vertigo attack(day), Median [IQR] | - | 18 [11 - 45] |  |  |  |
|  |  |  |  |  |  |
|  |  |  |  |  |  |

Supplementary Table 4. The clinical characteristics of cohort 4.

| Characteristic | Mean (SD) |  |  |  |  |
| --- | --- | --- | --- | --- | --- |
|  | MD (n=239) | Controls (n=35) | Statistic | *P* value |  |
| Age,Years | 52.4 (12.8) | 48.6 (13.1) | U=3670.5 | 0.242 |  |
| Female, No. (%) | 138 (57.7) | 18 (51.4) | χ2=0.496 | 0.481 |  |
| Left, No. (%) | 143 (59.8) | NA | NA | NA |  |
| Age of Oneset (SD) | 46.9 (13.9) | NA | NA | NA |  |
| Duration (min), mean (SD) | 279.8 (583.8) | NA | NA | NA |  |
| Migraine, No. (%) | 54 (22.6) | NA | NA | NA |  |
| Autoimmune disease, No. (%) | 7 (2.9) | NA | NA | NA |  |
| Allergy, No. (%) | 20 (8.4) | NA | NA | NA |  |
| Hypertension, No. (%) | 55 (23.0) | NA | NA | NA |  |
| Coronary Heart Disease, No. (%) | 12 (5.0) | NA | NA | NA |  |
| Diabetes, No. (%) | 18 (7.5) | NA | NA | NA |  |
| Smoking, No. (%) | 26 (10.9) | NA | NA | NA |  |
| PTA, dB | 56.4 (20.7) | NA | NA | NA |  |
| SDS (%) | 55.8 (28.4) | NA | NA | NA |  |
| UW (%) | 36.6 (23.0) | NA | NA | NA |  |
| vHIT VOR gain | 0.83 (0.24) | NA | NA | NA |  |
| cVEMP abnormal, No. (%) | 160 (66.9) | NA | NA | NA |  |
| oVEMP abnormal, No. (%) | 176 (73.6) | NA | NA | NA |  |
| Grading of endolymphatic hydrops, No. (%) |  |  |  |  |  |
| Normal | 33 (13.9) | NA | NA | NA |  |
| Mild | 52 (21.9) | NA | NA | NA |  |
| Severe | 152 (64.1) | NA | NA | NA |  |
| Data are shown as mean±SD. PTA, pure tone average of 0.5, 1 and 2 k Hz; SDS, speech discrimination score; UW, unilateral weakness; vHIT, video head impulse test; cVEMP, vestibular evoked myogenic potential; oVEMP, ocular vestibular evoked myogenic potential. NA, not applicable. | | | | |  |
|  |  |  |  |  |  |
|  |  |  |  |  |  |
|  |  |  |  |  |  |

Supplementary Table 5. Demographical characteristics of cohort 6.

| Characteristic | Mean (SD) |  |  |  |
| --- | --- | --- | --- | --- |
|  | MD (n=23) | Controls (n=26) | Statistic | *P* value |
| Age,Years | 56.3 (10.1) | 56.8 (6.5) | U=295 | 0.936 |
| Female, No. (%) | 9 (34.6) | 12 (52.2) | χ2=1.536 | 0.215 |

Supplementary Table 6. Primer sequences for quantitative RT-PCR.

| GENE | Forward primers (5’--3’) | Reverse primers (5’--3’) |
| --- | --- | --- |
| CCL24 | GGAGTGGGTCCAGAGGTACAT | CAGGTGGTTTGGTTGCCAG |
| CSF1 | TGGCGAGCAGGAGTATCAC | AGGTCTCCATCTGACTGTCAAT |
| CSF3R | GCGCGAGCAATAGCAACAAG | GTCACGATGATCTCATAGAGCTG |
| CXCR3 | CCACCTAGCTGTAGCAGACAC | AGGGCTCCTGCGTAGAAGTT |
| LTA | AGCACCCTAAGGCTGGACTT | CGGTTTTCCATGAACACATTCTC |
| IL6R | CCCCTCAGCAATGTTGTTTGT | CTCCGGGACTGCTAACTGG |
| TNFRSF1B | TTCATCCACGGATATTTGCAGG | GCTGGGGTAAGTGTACTGCC |
| IFI44 | GGTGGGCACTAATACAACTGG | CACACAGAATAAACGGCAGGTA |
| IFIT1 | GCGCTGGGTATGCGATCTC | CAGCCTGCCTTAGGGGAAG |
| IFIT5 | ACAAGTTGGAGTGTCCTGAGA | AAGCCGCTTTAGCCTTTTGATA |
| ISG15 | CGCAGATCACCCAGAAGATCG | TTCGTCGCATTTGTCCACCA |
| MXI1 | GCGCCTTTGTTTAGAACGCTT | AATGCTGTCCATTCGTATTCGT |
| TRIM23 | TGGTTGTAAACAAGCTCGGAG | ACTCTAGCACCTTCACTACAGC |
| ATF5 | TGGCTCGTAGACTATGGGAAA | ATCAACTCGCTCAGTCATCCA |
| NFKB1 | AACAGAGAGGATTTCGTTTCCG | TTTGACCTGAGGGTAAGACTTCT |
| RELA | GGGGACTACGACCTGAATG | GGGCACGATTGTCAAAGAT |
| STAT3 | ACCAGCAGTATAGCCGCTTC | GCCACAATCCGGGCAATCT |
| IRF2 | AATGCTGCCCCTATCAGAACG | CAGGACCGCATACTCAGGAGA |
| IRF9 | GCCCTACAAGGTGTATCAGTTG | TGCTGTCGCTTTGATGGTACT |
| CEBPB | CTGGAGACGCAGCACAAGGTCC | CTTGAACAAGTTCCGCAGGGTG |
| MAF | CTGGCAATGAGCAACTCCGA | AGCCGGTCATCCAGTAGTAGT |
| SPI1 | GTGCCCTATGACACGGATCTA | AGTCCCAGTAATGGTCGCTAT |
| KLF4 | CGGACATCAACGACGTGAG | GACGCCTTCAGCACGAACT |
| LEF1 | TGCCAAATATGAATAACGACCCA | GAGAAAAGTGCTCGTCACTGT |
| ADRA1A | CGCTACCCAACCATCGTCAC | GAACAGGGGTCCAATGGATATG |
| ADRA1B | TCTGGCGGTCATTCTAGTCAT | GGTGTCCTCGTGAAAGTTCTTG |
| ADRB1 | ATCGAGACCCTGTGTGTCATT | GTAGAAGGAGACTACGGACGAG |
| ADRB2 | TGGTGTGGATTGTGTCAGGC | GGCTTGGTTCGTGAAGAAGTC |
| ADRB3 | GACCAACGTGTTCGTGACTTC | GCACAGGGTTTCGATGCTG |
| IL6 | CCTTCGGTCCAGTTGCCTTCTCC | GCCAGTGCCTCTTTGCTGCTTTC |
| GCSF | TTAGAGCAAGTGAGGAAGATCC | CCATTCCCAGTTCTTCCATCT |
| TNFA | CCTCTCTCTAATCAGCCCTCTG | GAGGACCTGGGAGTAGATGAG |
| NR3C1 | ctgcctggtgtgctctgatgaag | taattgtgctgtccttccactgctc |
| NR3C2 | AGCAGCAGAACCAACAAGGAAG | CAACTTAGAGTGGAAGGACGATGG |

Supplementary Table 7. Assay specification for flow cytometer analysis.

| Cytokines (pg/ml) | Sensitivity Limit of Detection | Lower Limit of Quantification | Upper Limit of Quantification |
| --- | --- | --- | --- |
| G-CSF | <5 | <10 | >5,000 |
| IL-6 | <5 | <10 | >5,000 |
| TNF alpha | <1 | <2 | >1,000 |


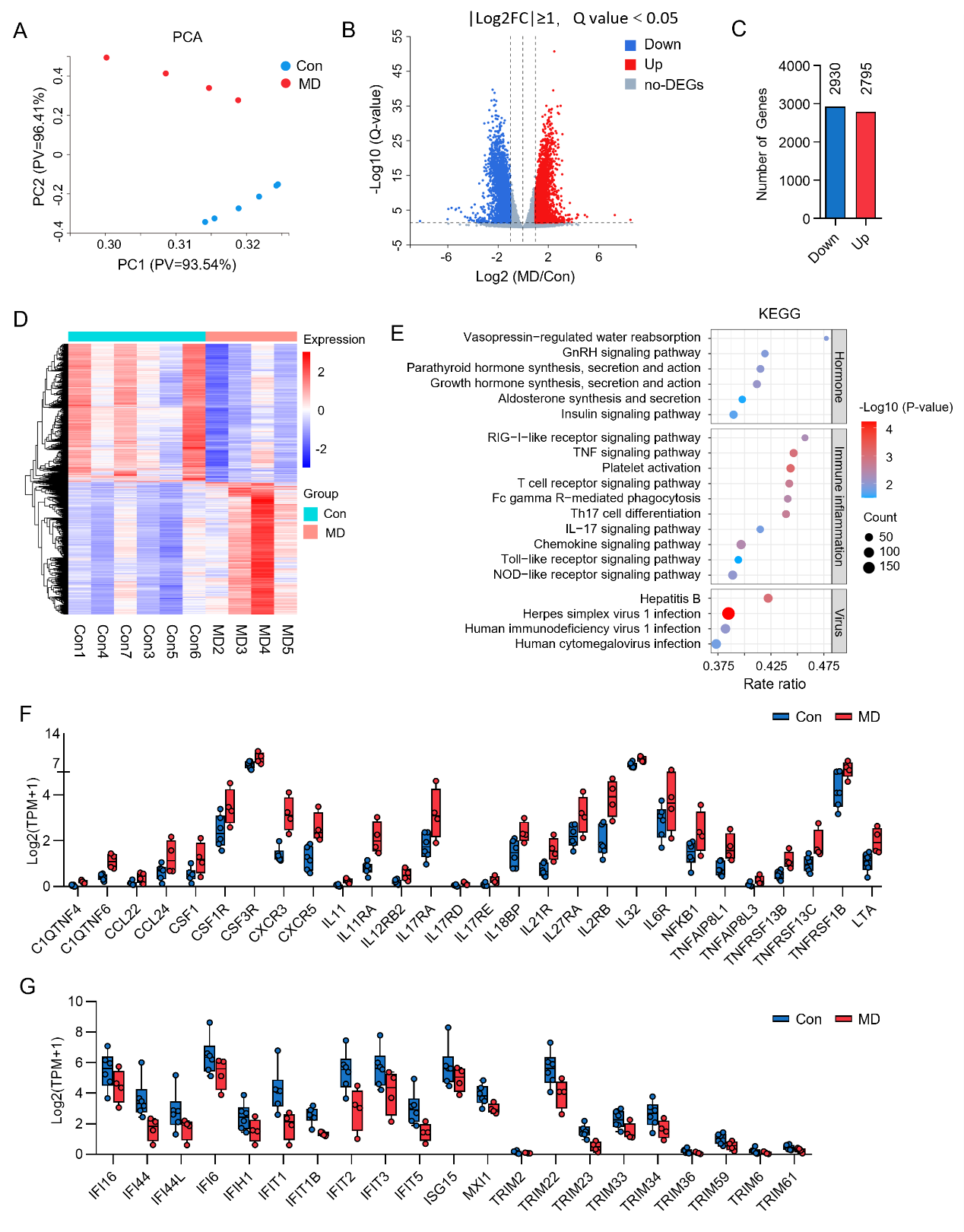


**Figure S1. Increased pro-inflammation genes and decreased anti-virus genes in MD**

A. Principal component analysis (PCA) results for PBMCS from controls and patients with MD. B. Volcano plot showing DEGs in the PBMCs (red, upregulated proteins; blue, downregulated proteins; grey, unchanged proteins). C. Histogram representing the distribution of DEGs in the PBMCs. D. Heat map showing the DEGs. E. Bubble diagrams of the KEGG pathway analysis. F. Box plot showing the TPM of gene expression distribution of control and MD samples. G. Box plot showing the TPM of gene expression distribution of control and MD samples.

Con, controls; MD, Ménière’s disease; DEGs; BP, biological process, cellular component; KEGG, Kyoto Encyclopedia of Genes and Genomes; GSEA, Gene set enrichment analysis.


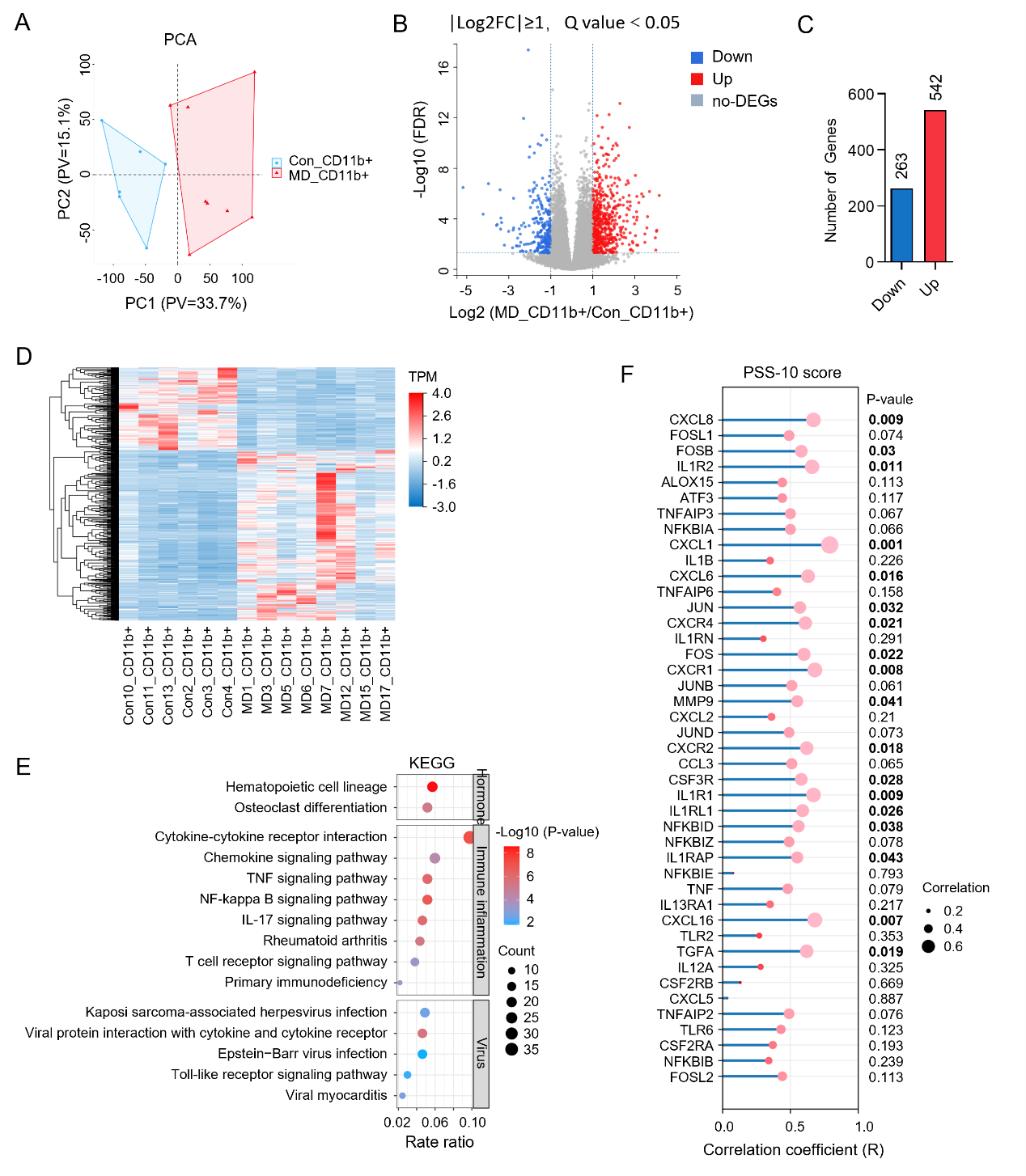


**Figure S2. CTRA cell origin and transcriptional regulation**

A. Principal component analysis (PCA) results for myeloid cells from controls and patients with MD. B. Volcano plot showing DEGs in myeloid cells (red, upregulated proteins; blue, downregulated proteins; grey, unchanged proteins). C. Histogram representing the distribution of DEGs in myeloid cells. D. Heat map showing the DEGs in myeloid cells. E. Bubble diagrams representing the enrichment analysis of the DEGs enriched in the KEGG pathway in myeloid cells. F. The transcriptional levels of most myeloid pro‑inflammatory genes were significantly positively correlated with PSS‑10 scores.

Con, controls; MD, Ménière’s disease.


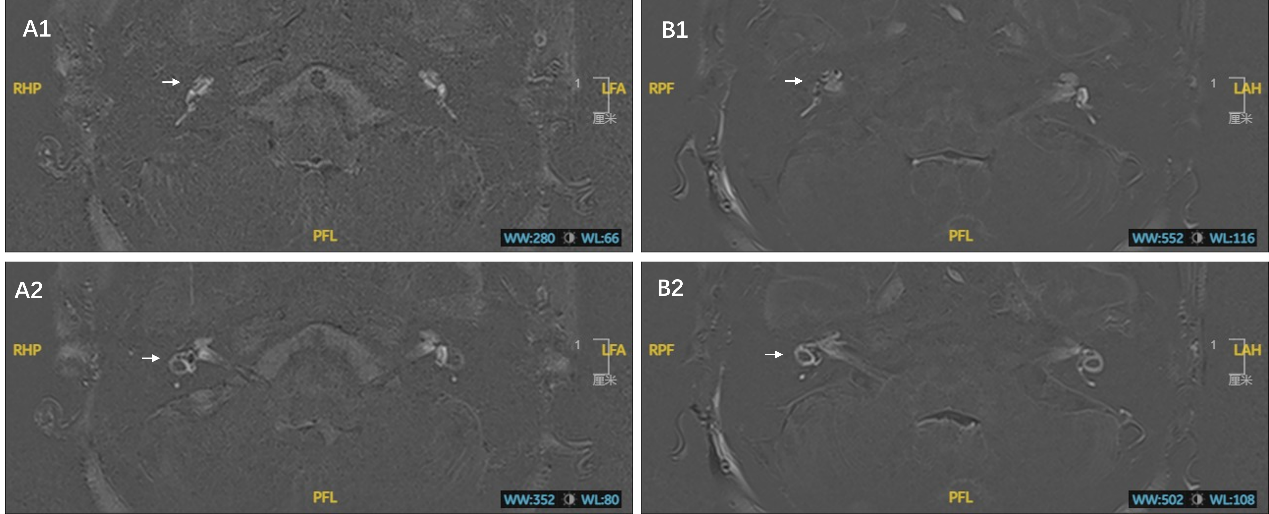


**Figure S3. Representative images of endolymphatic hydrops**. Panel A shows mild endolymphatic hydrops at the cochlear layer (A1) and vestibular layer (A2) in the affected ear (left side) of an MD patient. Panel B shows severe endolymphatic hydrops at the cochlear layer (B1) and vestibular layer (B2) in the affected ear (left side) of another MD patient. The contralateral (right) ears of both patients showed no endolymphatic hydrops. White arrow, lesion side; MD, Meniere’s disease.
